# ALDH3A1 acts as a prognostic biomarker and inhibits the epithelial mesenchymal transition of oral squamous cell carcinoma through IL-6/STAT3 signaling pathway

**DOI:** 10.7150/jca.40171

**Published:** 2020-02-19

**Authors:** Yi Qu, Ying He, Yang Yang, Shaoqing Li, Wei An, Zhilin Li, Xue Wang, Zhengxue Han, Lizheng Qin

**Affiliations:** 1 Medical Doctor, Department of Oral and Maxillofacial & Head and Neck Oncology, Beijing Stomatological Hospital, Capital Medical University, Beijing, China, 100050; 2 Professor and Medical Doctor, Department of Oral and Maxillofacial & Head and Neck Oncology, Beijing Stomatological Hospital, Capital Medical University, Beijing, China, 100050

**Keywords:** ALDH3A1, oral squamous cell carcinoma, lymph node metastasis, epithelial-mesenchymal transition, prognostic biomarker, IL-6

## Abstract

**Objectives:** Aldehyde dehydrogenase 3A1 (ALDH3A1) is a member of the ALDH superfamily and its relationship with oral squamous cell carcinoma (OSCC) still unknown. In our subject, we aimed to reveal the expression pattern and clinical value of ALDH3A1 in OSCC and its biological function in OSCC cell lines.

**Materials and methods:** The expression level of ALDH3A1 in paired OSCC tissues and adjacent noncancerous tissues were detected by quantitative real-time PCR, Western blot and immunohistochemistry. The relationship between ALDH3A1 expression and clinical characteristics was analyzed. Besides, cell-counting kit 8, colony formation, wound healing, transwell invasion, apoptosis and cell cycle assays were employed to assess the role of ALDH3A1 in OSCC cells. To explore the influence of ALDH3A1 on OSCC epithelial-to-mesenchymal transition (EMT), the expression of EMT markers (E-cadherin, vimentin, snail, MMP3) on OSCC cells were detected, and possible mechanisms were analyzed.

**Results:** In OSCC tissues, ALDH3A1 was significantly decreased compared to the adjacent normal tissues. Lower ALDH3A1 expression in OSCC tissues was associated with a higher incidence of lymph node metastasis (LNM). Moreover, the overall survival of OSCC with low ALDH3A1 expression was significantly worse compared to that of OSCC with high ALDH3A1 expression. Restored expression of ALDH3A1 suppressed cell proliferation, migration and invasion in OSCC cells. Further experiments showed that ALDH3A1 might inhibit EMT in OSCC via a regulation of the IL-6/STAT3 signal pathway.

**Conclusion:** These data indicate that ALDH3A1 may serve as a biomarker and may be developed into a novel treatment for OSCC.

## Introduction

Head and neck cancer is one of the most prevalent type of cancers with high mortality around the world, affecting more than 500,000 patients annually 1, 2. Oral squamous cell carcinoma (OSCC) accounts for 95% of all modalities of head and neck cancers 3. Surgery, with or without post-operative adjunctive therapy is recognized worldwide as the treatment of OSCC. In recent 30 years, there has been no significant improvement in survival outcome of OSCC, though many therapeutic advancements, with a 5-year survival rate around 60% 4. Besides, lymph node metastasis (LNM) is occurred in about 33% of OSCC patients 5. Neck nodal status of OSCC has consistently been shown to be independently associated with poor prognosis 6.

OSCC metastasis is a complicated process that involves isolation of cells from tumor tissue, regulation of cell activity, invasion, proliferation and evasion through the lymphatic system or blood vessels 7. In recent years, many studies reported that epithelial-to-mesenchymal transition (EMT) promotes epithelial-like tumor cells to acquire invasive potential, facilitates the transport of these cells through the blood or the lymphatic system 8. Various oncogenic pathways have been found to involve in this process of metastasis in different types of cancer including oral cancer 9, 10. Therefore, controlling the progression of EMT in OSCC is considered as a promising strategy to suppress metastasis and prolong patient survival. Duan et al. 11 addressed the function of CXCR4 in EMT in OSCC cells *in vitro* and in xenograft models, and found that CXCR4 silencing inhibited EMT in OSCC, thus reducing tumor metastasis. Iwata et al. 12 reported that percutaneous CO_2_ could improve hypoxia through suppressing EMT, leading to the reduction of the metastatic potential of OSCC.

Aldehyde dehydrogenase 3A1 (ALDH3A1) is an important member of the ALDH superfamily, which consists of NAD(P)^+^- dependent enzymes that oxidize a wide variety of endogenously produced and exogenously derived aldehydes to their corresponding carboxylic acids. In this way, they modulate several cell functions in normal and tumor cells, such as proliferation, differentiation, survival and response to oxidative stress 13, 14. ALDH3A1 is often overexpressed in esophagus - mucosa, minor salivary gland, nasal epithelium, tonsil and oral epithelium 15, 16. In recent years, ALDH3A1 has been shown to be upregulated in several cancer types and associated with poor clinical outcomes 17-19. Wu et al. 20 have demonstrated that ALDH3A1 is observably upregulated in stomach cancer stem-like cells, and high protein level of ALDH3A1 expression is associated with poor differentiation degree in stomach cancer tissues. Counihan et al. 21 used chemoproteomics-enabled covalent ligand screening to reveal that ALDH3A1 may be a new therapeutic target for lung cancer and that ALDH3A1 inhibitors damage the pathogenicity of lung cancer in cells with high expression of ALDH3A1. However, some studies have shown that not all tumors exhibit high levels of ALDH3A1 expression, and the correlation between ALDH3A1 over-expression and estimation of tumor progression or prognosis may not be significant 22, 23. Saiki et al. 24 indicated that higher ALDH3A1 protein levels in human head and neck cancer tumors were not associated with worse prognosis. Patel et al. 25 reported that ALDH3A1 is expressed at higher levels in normal lung tissues in comparison to small cell lung cancer tissue. To date, no study has attempted to reveal the expression pattern and biological functions of ALDH3A1 in OSCC.

In the present study, we found that ALDH3A1 expression was downregulated in OSCC tissues compared to their adjacent non-tumor tissues. Then the clinical implications of this phenomenon were further studied using an independent cohort of 72 paired OSCC and neighboring non-cancerous oral tissues. Further experiments showed that ALDH3A1 acts as a tumor suppressor gene and inhibits EMT via IL-6/STAT3 signaling pathway in OSCC cells.

## Materials and Methods

### Patients and tissue samples

An independent cohort study which comprised 72 OSCC patients who had a comparatively long follow up time was included in this study. A total of 72 pairs of OSCC and cancer‐adjacent normal tissues (ANTs) were collected at the Stomatology Hospital of Capital Medical University from January 2010 to December 2013. The study in accordance with Declaration of Helsinki and was approved by the Research Ethics Board of the Capital Medical University. The patients' inclusion criteria were as follows: (1) histopathological diagnosis of OSCC; (2) primary tumor resection and neck dissection were performed for treatment of OSCC without previous adjuvant therapy such as radiotherapy and/or chemotherapy. The exclusion criteria were as follows: (1) individuals who had other malignancies or a history of malignancy; (2) incomplete follow-up information. Clinicopathologic parameters were recorded including gender, age, TNM status and so on. After surgery, patients were followed up every 3-6 months until the fifth year. All the participants were informed of the nature of this study and signed the corresponding informed consent form.

One part of the collected tissues was immediately snap-frozen and stored in liquid nitrogen until use for quantitative real-time PCR and Western blot analyses. Another part of the specimens was immediately fixed in 4% buffered paraformaldehyde, routinely processed and embedded in paraffin for immunohistochemical staining.

### Quantitative reverse transcription (qRT)-PCR

Total RNA was prepared using TRIzol reagent following the manufacturer's instructions (CWbiotech, China). Next, we synthesized cDNA with a Prime Script RT Reagent Kit (Takara, Japan). Gene transcripts were quantified via real-time PCR performed with SYBR Green PCR kit (Qiagen, Germany). The sequences for the primers used were as follows: ALDH3A1, 5′- TGGAACGCCTACTATGAGGAG -3′ (forward) and 5′- GGGCTTGAGGACCACTGAG -3′ (reverse); IL-6, 5′- CCTGAACCTTCCAAAGATGGC -3′ (forward) and 5′- TTCACCAGGCAAGT CTCCTCA -3′ (reverse); STAT3, 5′- CAGCAGCTTGACACACGGTA -3′ (forward) and 5′- AAACACCAAAGTGGCATGTGA -3′ (reverse); β-actin, 5′- CATGTACGTTGCTATCCAGGC -3′ (forward) and 5′- CTCCTTAATGTCACG CACGAT -3′ (reverse). Gene expression was determined by the 2^-△△CT^ method.

### Western blot analysis

Western blot analysis was performed as previously described 26. The same amount of protein was separated by SDS gel and transferred to PVDF membranes. The membranes were blocked at room temperature in goat serum for 1 hour. And then each membrane was incubated with specific primary antibodies against ALDH3A1, E-cadherin and vimentin (Abcam, USA), p-STAT3 (Tyr705), MMP3 (Cell Signaling Technology, USA), IL-6, snail, STAT3 and GAPDH (ABclonal, USA) overnight. Next, the membranes were incubated with secondary antibodies (ABclonal, USA) and visualized using enhanced chemiluminescence reagents (Bio-Rad, USA).

### Immunohistochemical (IHC) staining

IHC was performed with 72 OSCC and paired ANT samples, as discussed previously 27. Briefly, the tissue sections were processed with antigen retrieval and blocked with goat serum. The sections were incubated with primary antibodies overnight at 4°C: rabbit anti-ALDH3A1 (1:200 dilution, Abcam, USA). After washing with PBS, the sections were incubated with HRP-labeled secondary antibody for 1 hour. Then the sections were sequentially incubated with streptavidin-peroxidase conjugate for 20 min and developing with 3, 3'-diaminobenzidine (DAB) as a chromogen substrate. The nuclei were stained with hematoxylin. The sections were mounted with a cover glass and evaluated under a microscope. A negative control without the primary antibody was run simultaneously.

The relative intensities of the completed immunohistochemical reactions were evaluated by 2 independent trained pathologists who were blinded to the subjects' clinical information. A scoring criterion for IHC was used in which the staining intensity and positive areas were recorded 28. Briefly, the intensity of anti-ALDH3A1 staining was scored according to the standards of 0 (no staining), 1 (weak staining), 2 (medium staining), and 3 (strong staining). The percentage of reactivity was scored as follows: 0 (no positive tumor cells), 1 (<10% positive tumor cells), 2 (10-40% positive tumor cells) and 3 (>40% positive tumor cells). The multiply of the two sub scores (range 0-9) was classified low expression (range 0-4) and high expression (range 5-9), respectively for statistical analysis. If there was a discrepancy in the score, it was regraded until a uniform conclusion was gained.

### Cell culture

The human oral epithelial cells (HOEC) were provided by the Wuhan University, (Wuhan, China). Human OSCC cell lines SCC15, SCC25 and Cal27 were obtained from American Type Culture Collection (ATCC, USA). The Tca83 cell lines was provided by the Capital Medical University (Beijing, China). HOEC and Tca83 cells were cultured in RPMI-1640 medium (Hyclone, USA). SCC25 and CAL27 cells were cultured in DMEM medium (Hyclone, USA). SCC15 was cultured in DMEM/F12 (1:1) medium (Hyclone, USA). All cells were supplemented with 10% FBS (Gibco, USA), 100 IU/mL penicillin and 100 mg/mL streptomycin (Beyotime Biotechnology, China).

### Lentiviral-mediated ALDH3A1 overexpression

Human ALDH3A1 lentiviral construct was generated by inserting the full-length human ALDH3A1 cDNA into the lentiviral vector (GeneChem, China). In order to establish stable ALDH3A1 overexpressed cell lines, SCC25 and SCC15 cells were transduced with lentiviral supernatant in the presence of 5 μg/ml polybrene and selected with 0.2 μg/mL puromycin for 10 days. ALDH3A1 expression was confirmed by qRT-PCR and Western blot analysis.

### Cell proliferation assay

Cell proliferation was analyzed using a Cell Counting Kit-8 (CCK-8) (Beyotime Biotechnology, China) according to the manufacturer's instructions. Briefly, 3000 OSCC cells were seeded in 96‐well plates and incubated for 5 days. At the appointed time, 10 μl of the CCK‐8 stock solution was added to each well and incubated at 37 °C for 2 h. The absorbance value was measured at 450 nm. All experiments were performed in triplicate.

### Colony formation assay

The transfected SCC15 cells (3000/well) and SCC25 cells (3000/well) were seeded on 6cm cell culture dishes, then further cultured for 10 days at 37°C. After incubation, the plate was washed twice with pre-cold PBS, stained with crystal violet, and the number of colonies was counted manually.

### Wound healing assay

An *in vitro* wound healing assay was used to assess cell motility. Briefly, the transfected SCC25 and SCC15 cells (1 X 10^6^/well) were seeded into 6-well plates. Once the cells reached 90% confluence, wounds scratching across the well were created by 200μl or 10μl pipette tips, from one end to the other end of the well and further cultured up to 72 hours. The monolayer was recorded at 10× magnification for calculating the movement speed of cells at 0, 24, 48 and 72 hours.

### Transwell cell invasion assay

Cell invasion was examined using transwell chambers (Corning, USA) with 8µm-pore polycarbonate filters. The SCC15 and SCC25 cells (5×10^5^ cells/ml) were suspended in serum-free DMEM/F12 and DMEM, and added to the upper chamber of the transwells. 750 μl of culture medium containing 10 % FBS was added into the lower chamber. A cotton swab was used to remove the cells that remained on the upper surface of the membrane after 48 h. The cells that penetrated across the polycarbonate membrane were fixed with 4 % formaldehyde for 15 min and stained with crystal violet for 15 min. Six random microscopic fields (×10 magnification) were photographed and counted.

### Annexin V/PE assays for apoptosis

The percentage of OSCC cell lines in early and late apoptosis was determined by AnnexinV-7-Amino-Actinomycin (7-AAD)/ Phycoerythrin (PE) staining according to the instruction (BD Biosciences, USA). Annexin V+/PE- staining and Annexin V+/PE+ staining were used to define early apoptosis and late apoptosis.

### Cell cycle analysis

We used the Cell Cycle Analysis Kit (Beyotime Biotechnology, China) for cell cycle analysis. Briefly, cells were fixed in 70% ethanol at 4^◦^C for 24 h. After fixation, cells were washed and stained with 0.5 ml of propidium iodide (PI) staining buffer at 37 ^◦^C for 30 min in the dark. After incubation, red fluorescence was detected at an excitation wavelength of 488 nm and the percentage of cells at the G0/G1, S, and G2/M phases were quantified by the ModFit software. The experiments were repeated for three times.

### Statistical analysis

All data were evaluated using IBM SPSS version 21.0. Student's t-test or one-way analysis of variance (ANOVA) test was performed to analyze the significance of differences between samples obtained from three independent experiments. Clinical associations between ALDH3A1 mRNA expression and clinicopathological parameters were compared by Student's t-test. Moreover, the relationship between ALDH3A1 protein expression and clinicopathological parameters was compared by Chi-square test. Survival analyses were conducted by the Kaplan-Meier method, and curves were compared by the log-rank test. Cox proportional hazard model was used to explore all prognostic factors by univariable analysis and effects of confounding factors were adjusted for using multivariate analysis. The quantitative data in this study were expressed as the means ± standard deviation (SD). Differences were considered significant at the value of P < 0.05.

## Results

### Expression levels of ALDH3A1 in tissues and cell lines

**Figure 1:**
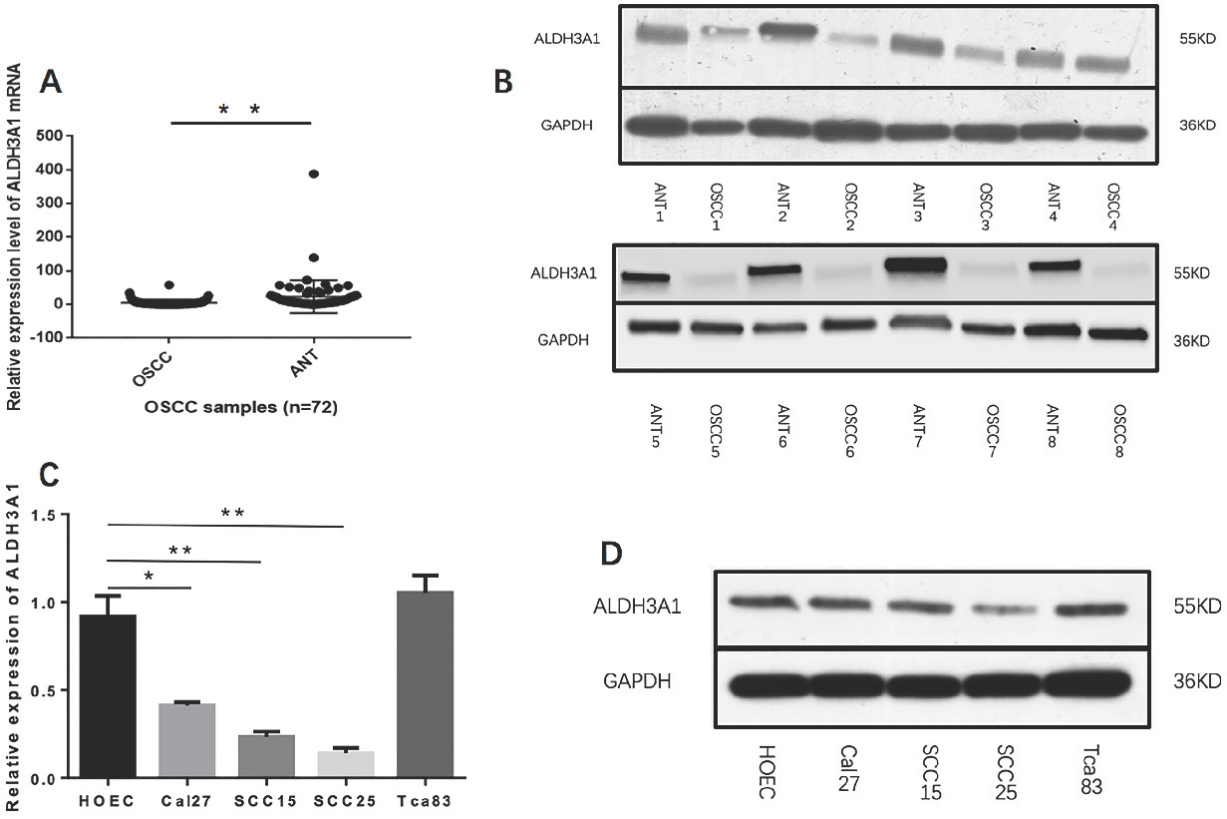
ALDH3A1 expression in tissues and cell lines. (A) qRT-PCR analysis of ALDH3A1 expression in OSCC samples and cancer‐adjacent normal tissues (ANTs). (B) Western blot analysis of ALDH3A1 protein levels in 8 OSCC samples and paired adjacent normal tissue (ANT) samples. (C-D) mRNA and protein expression of ALDH3A1 in HOEC and OSCC cell lines, including SCC15, SCC25, Cal27 and Tca83. *p< 0.05, **p< 0.01.

We first measured the expression levels of ALDH3A1 in 72 pairs of OSCC and matched ANT samples. According to the qRT-PCR results, ALDH3A1 mRNA levels were notably downregulated in OSCC tissues compared with paired ANTs (Figure 1A, 5.08±8.91 versus 23.28±48.54, P<0.01). We also detected the protein expression of ALDH3A1 by Western blot analysis; the results indicated that ALDH3A1 expression was reduced in the vast majority of detected tumor samples compared with the ANTs from the same patient (Figure 1B).

Moreover, we evaluated the expression levels of ALDH3A1 in SCC15, SCC25, Cal27, Tca83 and HOEC. Observably, ALDH3A1 mRNA expression in the SCC15, SCC25 and Cal27 cell lines were lower than in HOEC. Furthermore, there was no statistically significant difference in ALDH3A1 mRNA expression levels between Tca83 and HOEC (Figure 1C). Western blot analysis shows the same results (Figure 1D). To avoid differences between cell lines, we chose both SCC15 and SCC25 for further experiments because they showed the lowest expression of ALDH3A1. Our results demonstrated that ALDH3A1 was downregulated in OSCC tissues and SCC15, SCC25, Cal27 cells compared to corresponding ANTs and HOEC.

### The correlation between ALDH3A1 expression and clinicopathological parameters

**Figure 2:**
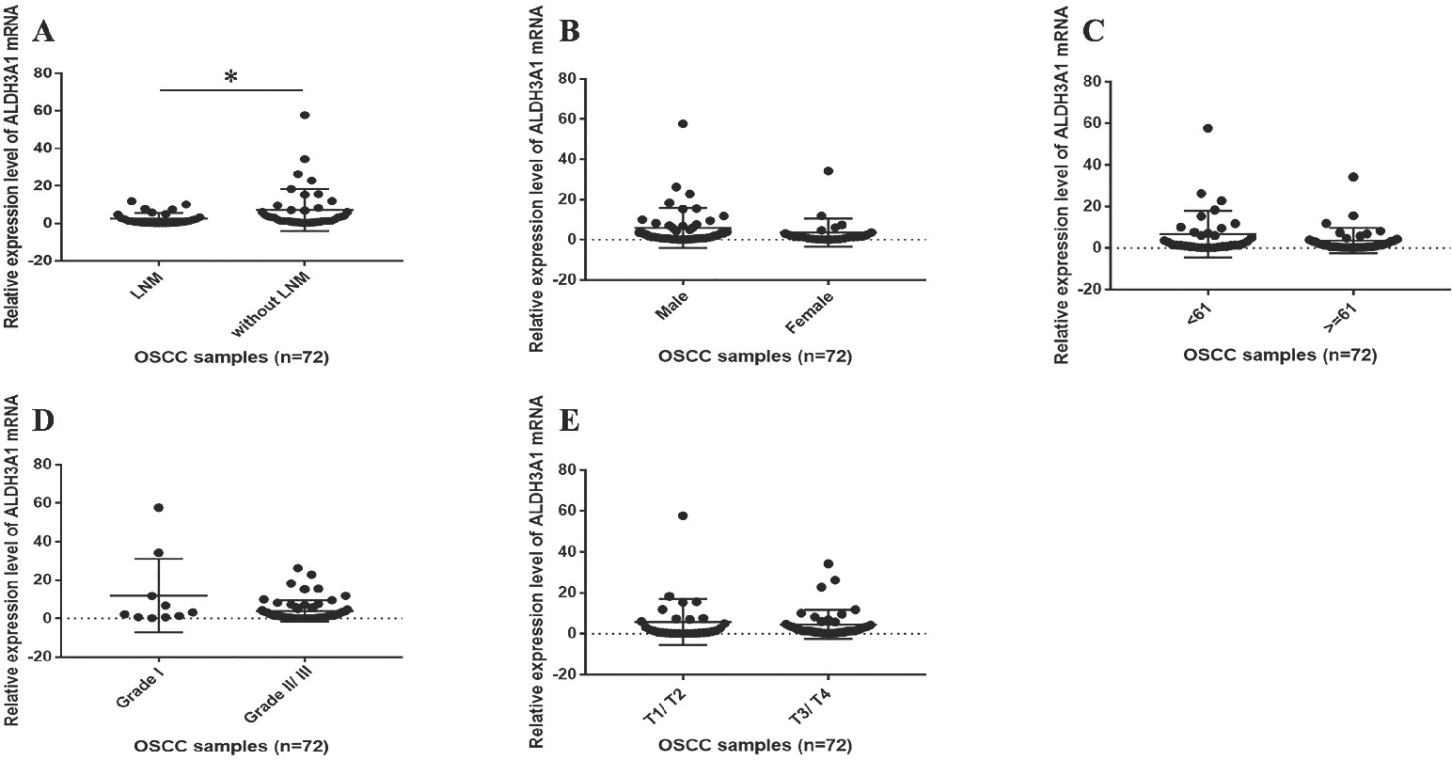
The correlation between ALDH3A1 mRNA expression levels and clinical features in OSCC patients. (A) The mRNA expression levels of ALDH3A1 in tissues from OSCC patients with and without LNM. *p< 0.05. (B) The mRNA expression levels of ALDH3A1 in tissues from male and female OSCC patients. (C) The mRNA expression levels of ALDH3A1 in tissues from < 61 years and ≥ 61 years OSCC patients (61 years is the median age of the subjects). (D) The mRNA expression levels of ALDH3A1 in tissues from T1/T2 and T3/T4 OSCC patients. (E) The mRNA expression levels of ALDH3A1 in tissues from histology grade I and grade II/III OSCC patients.

We subsequently assessed whether ALDH3A1 could pinpoint specific molecular signatures with clinical relevance in OSCC. Seventy-two patients were included in this research. The female-to-male ratio was 1:1.9, with an average age of 61.5±10.1 years (range from 42 to 83 year). The mean follow-up was 47.6±19.4 months. Five types of clinical and pathological features (age, gender, clinical T stage, histology grade and neck nodal stage) were analyzed. In OSCC patients with LNM, the mRNA expression levels of ALDH3A1 were statistically lower than those without LNM (Figure 2A, Table 1). However, no statistical significance was observed when ALDH3A1 expression was stratified according to sex, age, histology grade and clinical T stage in OSCC tissues (Figure 2B, 2C, 2D, 2E, Table 1).

**Figure 3:**
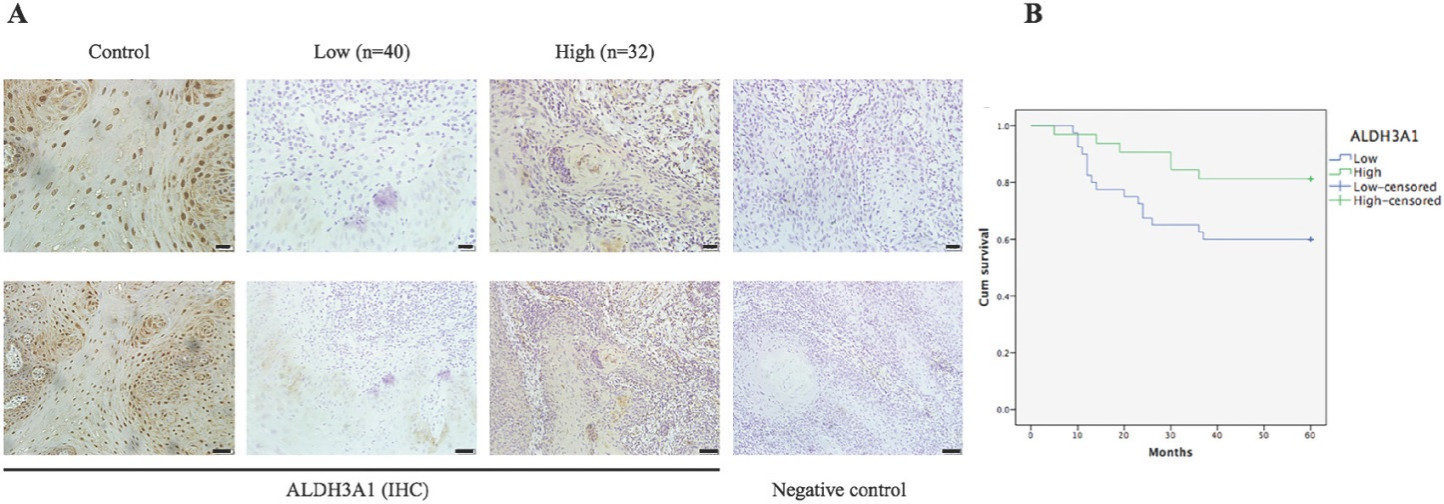
IHC results for the expression of ALDH3A1 and its relationship with overall survival in patients with OSCC. (A) IHC staining of ALDH3A1 in control (adjacent normal tissues) and OSCC tissues. The negative control was established by using PBS as a substitute for the primary antibody. Scale bar: 20 μm (top) and 50 μm (bottom). (B) Kaplan-Meier survival analysis was performed to estimate the association between ALDH3A1 protein expression levels in tissue and the survival time of OSCC patients (p=0.046).

According to immunoreactivity score (IS) of ALDH3A1, 72 OSCC patients were divided into two groups. Figure 3A shows ALDH3A1 expression in different tissues. ALDH3A1 was mainly expressed in the cytoplasm of the ANTs, showing obvious pale brown. However, in the cancer tissue, ALDH3A1 was less expressed at different levels, even in some cases, ALDH3A1 expression totally disappeared. The results showed that 55.6% (40/72) of OSCC tissues displayed lower expression of ALDH3A1. Chi-squared test was used to analyze the relationship between ALDH3A1 protein expression level and clinicopathologic features. As shown in Table 2, ALDH3A1 levels were significantly correlated with LNM (P=0.037). Patients in the low-ALDH3A1 group were more likely to have LNM. There was no association between ALDH3A1 protein expression levels and gender, age, clinical T stage or pathological grade. The results of ALDH3A1 protein expression level were consistent with those of mRNA expression level.

All patients were divided into two groups according to IS to investigate the importance of ALDH3A1 in predicting clinical outcomes of OSCC patients. Kaplan-Meier analysis showed that the total survival time of patients with a lower ALDH3A1 expression was significantly shorter than that of patients with a higher ALDH3A1 expression (Figure 3B, P=0.046). In the univariate analysis, LNM and ALDH3A1 in tissues were both risk factors for patient prognosis. However, in the multivariate analysis, only LNM was the significantly independent predictor for OSCC patients (Table 3).

### Overexpression of ALDH3A1 inhibits cell proliferation in OSCC cells *in vitro*

**Figure 4:**
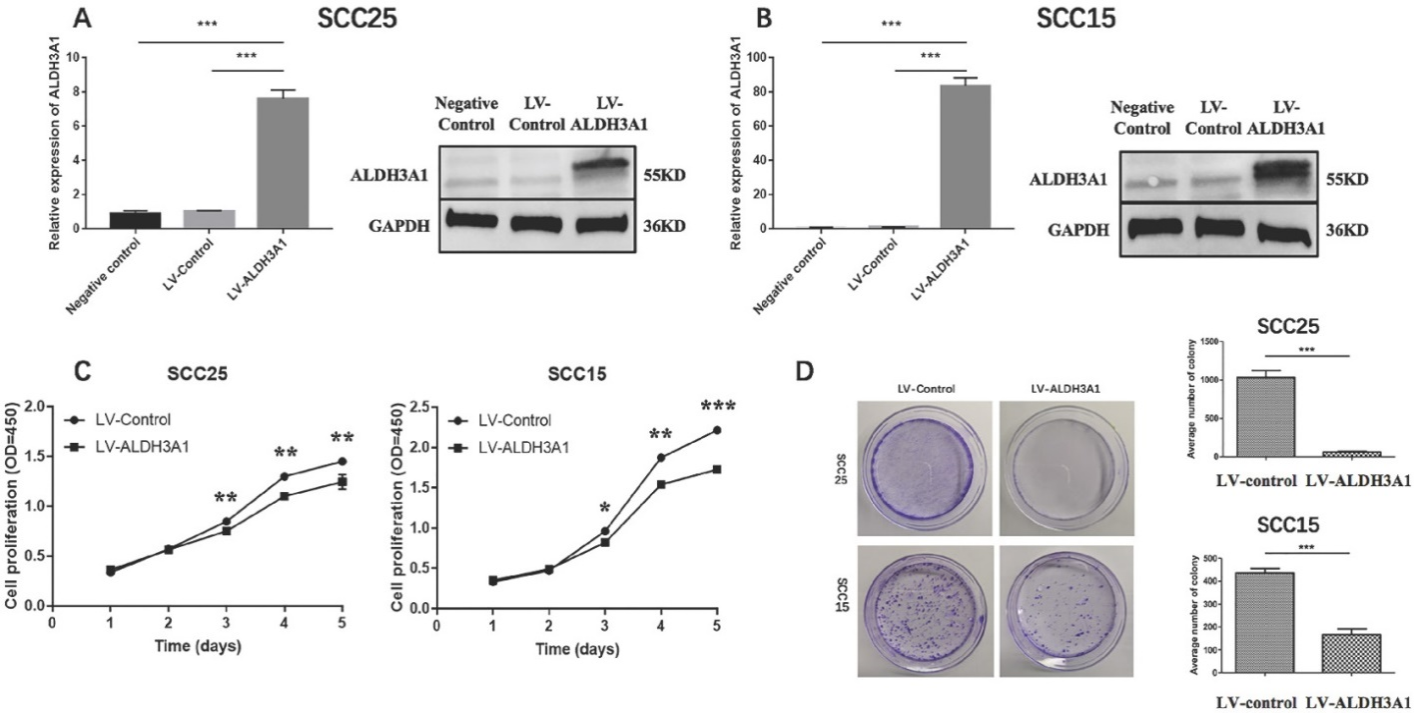
ALDH3A1 inhibits cell proliferation and colony‐formation abilities in SCC15 and SCC25 cells. (A-B) qRT‐PCR and Western blot results for SCC25 and SCC15 transfected with control lentivirus and ALDH3A1 lentivirus. (C) Cell proliferation was determined by CCK-8 assay at 1, 2, 3, 4 and 5 days. (D) Colony formation assay results. Quantification analyses for D; the data are the mean ± SD of colony numbers. *p< 0.05, **p< 0.01, ***p<0.001.

In order to study the characteristics of ALDH3A1 in OSCC cells, the following functional studies of ALDH3A1 were conducted on SCC15 and SCC25 cell lines. We generated stable SCC15 and SCC25 cells transfected with lentivirus for overexpressing ALDH3A1. Compared with control cells, ALDH3A1 mRNA and protein expression levels were increased in ALDH3A1-vector-transfected SCC15 and SCC25 cell lines (Figure 4A, 4B). Thus, we used these stable cell lines for further study. The proliferation of SCC15 and SCC25 cells overexpressing ALDH3A1 was significantly inhibited at 3, 4, and 5 days as measured by the CCK-8 assay (Figure 4C). Additionally, we conducted a colony formation experiment to further demonstrate the ability of cell proliferation. ALDH3A1-vector-transfected SCC15 and SCC25 cell lines, control-vector -transfected SCC15 and SCC25 cell lines were seeded in 6cm cell culture dishes, then photographed after 8 days of growth. A markedly lower number of colonies for the ALDH3A1-overexpressing cells than the control cells by counting the number of colonies for each group have been found (Figure 4D). In conclusion, our results illuminate the potential role of ALDH3A1 in inhibiting cell proliferation *in vitro*.

### ALDH3A1 overexpression inhibits the migration and invasion of OSCC cells

**Figure 5:**
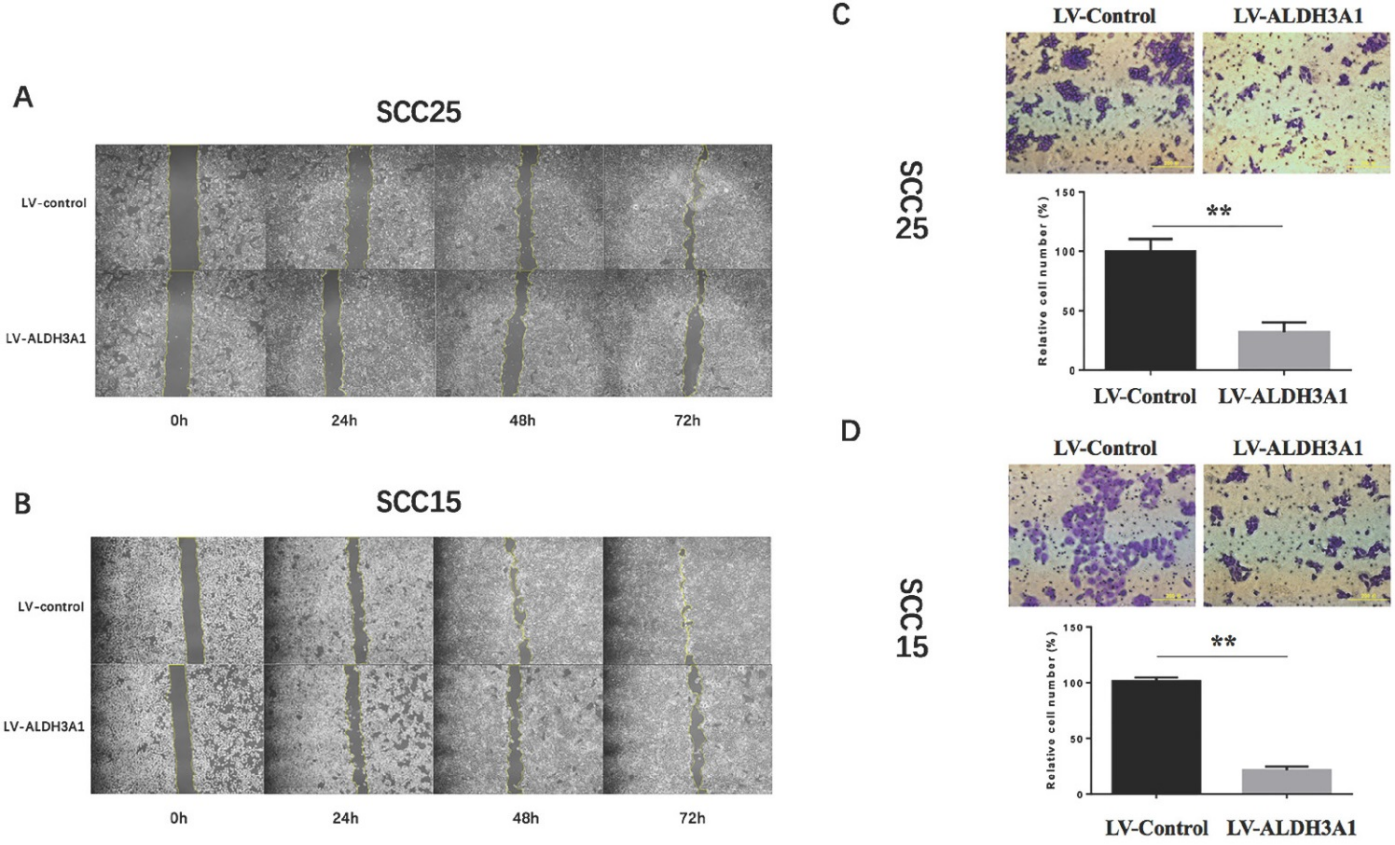
Up-regulation of ALDH3A1 inhibited cell migration and metastasis *in vitro*. (A-B) Up-regulation of ALDH3A1 inhibited migration in SCC15 and SCC25 cells at 48h and 72h, in a wound-healing assay. The representative images were from at least three independent experiments. (C-D) Up-regulation of ALDH3A1 inhibited SCC15 and SCC25 cell invasion in a cell invasion assay, 100× magnifications. The representative images were from at least three independent experiments. **p< 0.01.

To determine whether ALDH3A1 plays a role in OSCC cell migration *in vitro*, we conducted a wound healing assay. Up-regulation of ALDH3A1 decreased migration in SCC15 and SCC25 cells at 48h and 72h (Figure 5A, 5B). Since metastasis is the most aggressive function of cancer cells, transwell invasion assay was used to detect the metastatic ability of transfected OSCC cells. According to the numerical scoring, up-regulation of ALDH3A1 in OSCC cell lines significantly reduced the number of invasive cells after 48 h of incubation in invasion chambers (Figure 5C, 5D).

### Effects of ALDH3A1 on OSCC apoptosis and cell cycle

**Figure 6:**
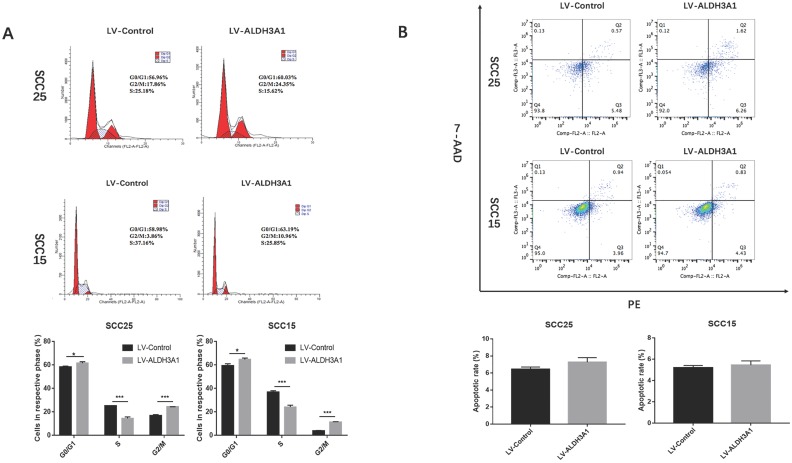
Effects of ALDH3A1 on OSCC cell cycle and apoptosis. (A) Representative plots of the cell cycle phases are shown for the SCC15 and SCC25 cells. The representative images were from at least three independent experiments. (B) The cell apoptosis distribution after transfection was detected by flow cytometry. The representative images were from at least three independent experiments. *p< 0.05, **p< 0.01, ***p<0.001.

Cell cycle analysis was used to investigate the effects of ALDH3A1 on the growth of SCC15 and SCC25 cells (Figure 6A). The results indicated that overexpression of ALDH3A1 decreased the percentage of OSCC cells in S phase and increased the percentage of cells in G0/G1 phase.

In addition, the early and late cell apoptosis ratios of SCC15 and SCC25 cells were examined (Figure 6B). There was no statistical significance in the apoptosis rate when the cells were transfected with ALDH3A1 lentivirus compared to that of their respective controls in both SCC15 and SCC25 cells (P>0.05).

### ALDH3A1 suppresses epithelial-mesenchymal transition by targeting the IL-6/STAT3 axis in OSCC cells

**Figure 7:**
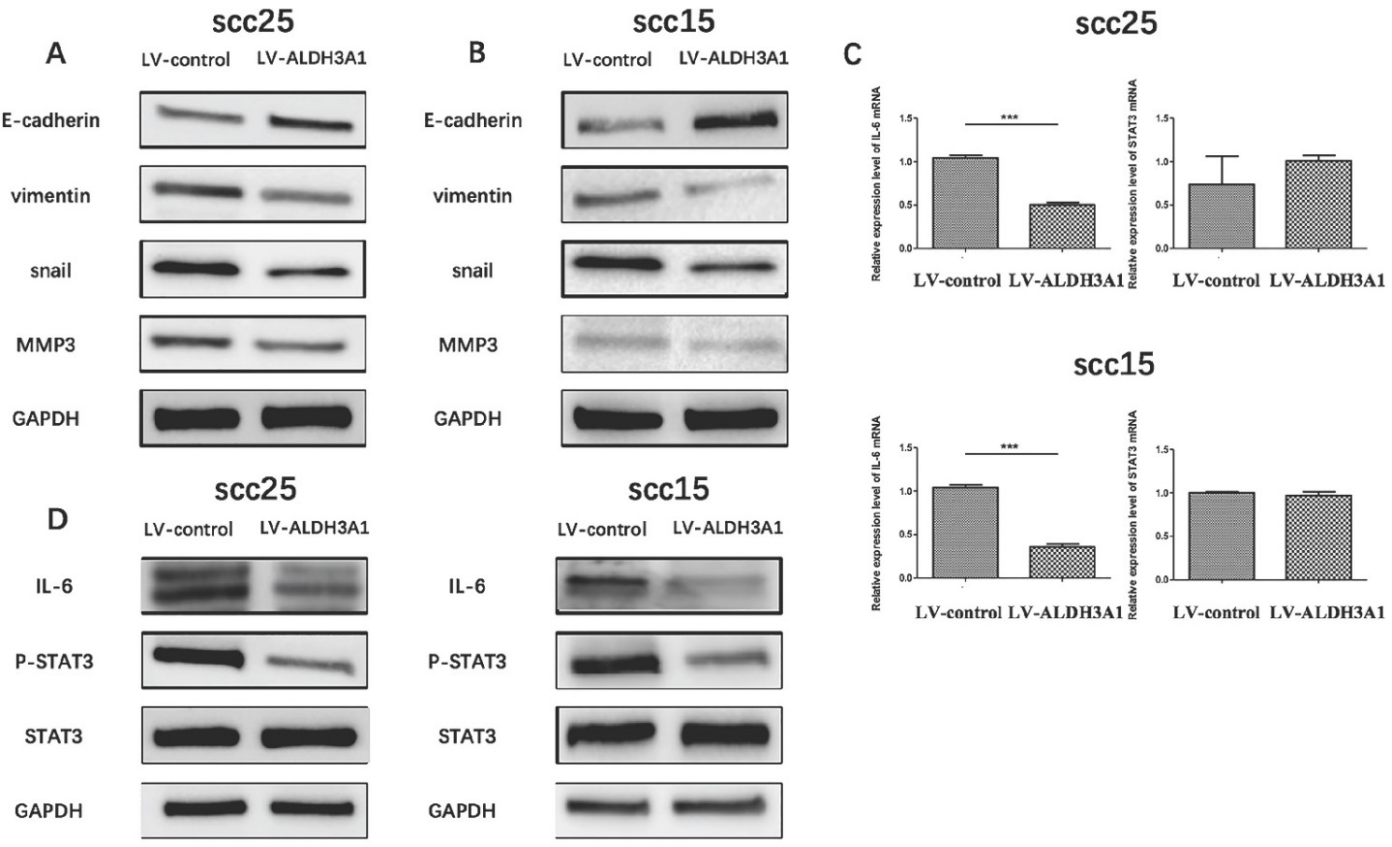
ALDH3A1 suppresses EMT by targeting the IL-6/STAT3 axis in OSCC cells (A-B) The expression level of E-cadherin, vimentin, snail and MMP-3 protein expression in SCC15 and SCC25 cells were examined by western blot analysis. (C) The mRNA expression levels of IL-6 and STAT3 in SCC25 and SCC25 cells. (D) Western blot of phosphorylated STAT3 (Tyr705), STAT3 and IL-6 in SCC15 and SCC25 cells. ***p<0.001.

The important role that EMT plays in tumor invasion and metastasis has been well verified 29, 30. To further investigate the influence of ALDH3A1 on OSCC cell EMT, the expression of EMT markers (E-cadherin, vimentin, snail, MMP3) on SCC15 and SCC25 cells were detected using western blot analysis. As illustrated in Figure 7A and 7B, E-cadherin expression was significantly increased, although vimentin, snail and MMP3 expression were suppressed with ALDH3A1 overexpression in SCC15 and SCC25 cells as compared with the control group.

The role of Interleukin-6 (IL-6) in EMT induction has been demonstrated 31. In addition, recent studies have revealed the important role of STAT3 in IL-6-modulated EMT 32. To further investigate the potential mechanism of ALDH3A1 on OSCC cell EMT, qRT-PCR and Western blot analysis were used to determine the impact of ALDH3A1 overexpression on the IL-6/STAT3 signaling pathways. IL-6 mRNA expression levels were decreased in ALDH3A1-vector-transfected SCC15 and SCC25 cells compared with the control cells (Figure 7C). Furthermore, STAT3 phosphorylation and IL-6 protein expression were suppressed in ALDH3A1-overexpressing cells compared with the control cells (Figure 7D). Collectively, these results revealed that ALDH3A1 suppresses EMT in OSCC cells, and the IL-6/STAT3 signaling pathway may be involved in this process.

## Discussion

Among all subtypes of oral malignancies, more than 90% are comprised of OSCC, which is a characteristic tumor with local aggressiveness 3. Although, there are various advanced treatment options, local or regional relapse and cervical LNM are still threats to patients 4. Therefore, new highly sensitive and specific biomarkers are required for more effective treatment of this disease.

ALDH3A1, a member of the ALDH family, performs a variety of biological functions including maintaining hematopoietic stem cells, protecting the eye from ultraviolet radiation, regulating cell proliferation and so on 13, 33. Recently, many studies have revealed that ALDH3A1 can be used as a biomarker to predict the prognosis of malignant tumors, and have been associated with different clinical outcomes in a wide variety of human malignancies 17-21. However, whether ALDH3A1 can also be used as a biomarker to predict the prognosis of patients with OSCC has not been studied.

In this subject, we present the first evidence of ALDH3A1 mRNA and protein expression in OSCC, indicating that ALDH3A1 was significantly downregulated in OSCC tissues compared with the paired ANTs. The reason for these results may be that ALDH3A1 is overexpressed in normal oral epithelium. In addition, OSCC originates from the upper layer of oral mucosa, which is different from other malignant tumors. To explore the clinical significance of ALDH3A1 in OSCC, qRT-PCR and IHC were performed using 72 OSCC tissues. The results indicated that a lower ALDH3A1 expression in OSCC was related to a higher incidence of LNM. Moreover, the prognosis of OSCC with low ALDH3A1 expression was significantly worse compared with that of OSCC with high ALDH3A1 expression, though multivariate analysis did not reveal ALDH3A1 expression as an independent prognostic factor. The possible reasons for this result may due to the small sample size. More importantly, the association between low ALDH3A1 expression and the poorer overall survival of OSCC patients was mainly through its association with a higher rate of LNM. These data suggest that ALDH3A1 may be a novel biomarker to predict LNM and prognosis of OSCC patients.

To analyze the function of ALDH3A1 in OSCC cells, we first investigated the ALDH3A1 mRNA and protein expression in HOEC, SCC15, SCC25, Cal27 and Tca83 cells. The results showed that ALDH3A1 expression was low in SCC15 and SCC25 cells. The overexpression of ALDH3A1 in SCC15 and SCC25 cell lines markedly inhibited cell growth, colony formation ability, migration, and invasion. To investigate the cellular mechanism underlying the anti-proliferative effects of ALDH3A1 in OSCC cells, cell cycle distribution and cell apoptosis were analyzed. The results found that ALDH3A1 overexpression could arrest cell cycle progression at the G1 phase, but not induce cell apoptosis in SCC15 and SCC25 cells. These findings reveal that ALDH3A1 may play a tumor suppressor role in OSCC.

EMT is an important prognostic factor in OSCC and is involved in early recurrence or metastasis after surgery 34, 35. Currently, blocking EMT is considered as a promising strategy to inhibit cancer metastasis and improve patient survival. In order to investigate the influence of ALDH3A1 on OSCC cell EMT, the expression of EMT markers (E-cadherin, vimentin, snail, MMP-3) were detected using western blot analysis. Notably, Snail is a zinc finger transcription factor which has been shown to regulate the transcriptional inhibition of E-cadherin 36. MMP-3 could cleave several component proteins of the extracellular matrix to promote migration and invasion and induce EMT 37. The results found that overexpression of ALDH3A1 could lead to significant upregulation of the epithelial marker (E-cadherin) and downregulation of the mesenchymal marker (vimentin), as well as snail and MMP-3 in SCC15 and SCC25 cells.

Proinflammatory cytokine IL-6 is a primary cytokine in the tumor microenvironment, which is produced by macrophages, neutrophils, monocytes, tumor cells and other cell types 38-40. IL-6 has been demonstrated to promote the invasion of blood vessels by cancer cells through its role as an important trigger of EMT 41. Studies have shown that the STAT3 pathway is involved in IL-6 regulation of several cancer cell functions, such as proliferation and metastasis 42. Notably, STAT3 has been demonstrated to play a key role in IL-6-induced EMT 43. In this study, we found that the expression of IL-6 and phosphor-STAT3 were suppressed in ALDH3A1-overexpressing cells. Therefore, these results demonstrated that upregulation of ALDH3A1 could effectively reverse the IL-6 induced EMT of OSCC cells through the IL6/STAT3 axis.

## Conclusion

Our study strongly supports the use of ALDH3A1 as a biomarker to predict lymph node metastasis and prognosis of OSCC patients, and suggests that it could function as a tumor suppressor in OSCC cells. Furthermore, identifying dysregulated ALDH3A1 expression in OSCC cells will help us better understand tumor progression and metastasis, and suggest a possible therapeutic strategy for oral cancer.

## Figures and Tables

**Table 1:** Relationship between ALDH3A1 mRNA expression and the clinicopathological characteristics of the 72 OSCC patients

Variables	All cases		ALDH3A1 ^2-△△CTb^	P-value
	No.	%	Mean ± SD	
**Age^a^**				0.145
<61	34	47.2	6.71 ± 11.06	
≥61	38	52.8	3.62 ± 6.04	
**Gender**				0.238
Male	47	65.3	5.91 ± 9.74	
Female	25	34.7	3.52 ± 6.82	
**Clinical T stage**				0.604
T1/T2	29	40.3	5.82 ± 11.07	
T3/T4	43	59.7	4.58 ± 7.05	
**Pathological grade**				0.223
I	10	13.9	11.91 ± 18.12	
II/III	62	86.1	3.98 ± 5.52	
**Lymph node metastasis**				0.015*
Absent	42	58.3	7.09 ± 11.09	
Present	30	41.7	2.43 ± 3.07	

Abbreviations: SD, standard deviation. *P<0.05. a: 61 years is the median age of the subjects. b: 2^-△△CT^ indicates the difference in the cycle number at which a sample's fluorescent signal passes a given threshold above baseline (Ct) derived from a specific gene compared with that of β-actin in tumor tissues.

**Table 2:** Correlation of ALDH3A1 protein expression and pathological characteristics of the 72 OSCC patients

Variables	All cases	ALDH3A1		
		Low	High	P-value^a^
**Age**				0.37
<61	34	17	17	
≥61	38	23	15	
**Gender**				0.58
Male	47	25	22	
Female	25	15	10	
**Clinical T stage**				0.667
T1/T2	29	17	12	
T3/T4	43	23	20	
**Pathological grade**				0.286
I	10	4	6	
II/III	62	36	26	
**Lymph node metastasis**				0.037^*^
Absent	42	19	23	
Present	30	21	9	

*P<0.05. a: Chi-square test.

**Table 3:** Univariate and multivariate analyses on survival in OSCC patients

Characteristics	Subset	Hazard ratio (95% CI)	p-value
Univariate analysis			
Gender	Female/Male	0.594 (0.257-1.375)	0.224
Age	Age≥61/Age<61	1.458 (0.623-3.413)	0.384
Clinical T stage	T1, T2/T3,T4	1.260 (0.529-3.005)	0.602
Pathological grade	I/II, III	3.839 (0.516-28.547)	0.189
Lymph node metastasis	absent/present	12.098 (3.565-41.060)	0.0064^**^
ALDH3A1	Low/High	0.399 (0.156-1.020)	0.045^*^
Multivariate			
Lymph node metastasis	absent/present	12.098 (3.565-41.060)	0.0064^**^

*P < 0.05 and **P < 0.01.
